# Physicochemical Properties of Sprouted Fava Bean Flour–Fermented Red Rice Flour Mixed System and Its Application in Gluten-Free Noodles

**DOI:** 10.3390/foods14244302

**Published:** 2025-12-14

**Authors:** Zhongman Min, Ting Zheng, Jinghan Wang, Wenkai Hu, Qingyu Yang, Yanwen Kong

**Affiliations:** College of Grain Science and Technology, Shenyang Normal University, Shenyang 110034, China; sy776838@icloud.com (Z.M.); zhengting130@163.com (T.Z.); 18648165720@163.com (J.W.); 18220023061@163.com (W.H.)

**Keywords:** fermentation, germination, dough interactions, noodle quality, functional properties

## Abstract

This study investigated the functional, pasting, and rheological properties of sprouted fava bean flour, *Lactobacillus plantarum* fermented red rice flour, and mixed flour. Then, the study investigated the structural properties of sprouted fava bean dough, fermented red rice dough, and mixed dough, as well as the quality and functional properties of the mixed noodles. The results showed that sprouting and fermentation treatments improved the processing properties of the flour. X-ray diffraction (XRD), Fourier transform infrared spectroscopy (FTIR), and moisture distribution results of the dough showed that the mixed dough had a more stable structure. Scanning electron microscopy (SEM) and Confocal laser scanning microscopy (CLSM) of the noodles showed that the addition of sprouted fava bean flour was able to mimic the protein network structure to some extent. In addition, germination and fermentation were effective in improving the textural properties, as well as increasing the total phenolic content (1.27–2.03 mg GAE/g) and antioxidant activity, including 1,1-diphenyl-2-picrylhydrazyl (DPPH) radical scavenging (43.54–64.84%), 2,2′-azinobis-(3-ethylbenzothiazoline-6-sulfonic acid) (ABTS) radical scavenging (41.37–58.98%), Hydroxyl radical scavenging (19–48.38%), while significantly reducing the estimated glycemic index (74.64–57.60) of the mixed noodles.

## 1. Introduction

Celiac disease is an autoimmune intestinal disorder caused by patients’ intolerance to gluten proteins in wheat, barley, and other grains [1]. Consuming gluten-containing foods may cause damage to the small intestine, leading to symptoms such as diarrhea, abdominal pain, and anemia [2]. Therefore, it is important to develop gluten-free products. Noodles have become a widely chosen staple food in Asia due to their diversity and ease of preparation. However, traditional noodles are typically made from wheat flour. For people with gluten sensitivities, it is necessary to look for non-wheat flours such as rice flour or legume flour to replace traditional wheat noodles.

Currently, commercially available gluten-free noodles are usually made from polished rice flour. However, refined rice has a high glycemic index [3]. Red rice, as a colored rice, has received much attention from researchers and consumers for its unique health benefits, and thus has been used in many food formulations (e.g., noodles [4], bakery products [5], breakfast cereal [6]). Studies have shown that red rice not only has a lower glycemic index than white rice, but also has a higher content of dietary fiber, which contributes to glycemic control [7]. In addition, the bran layer of red rice grains is rich in antioxidant bioactive components such as phenolic acids, flavonoids, and anthocyanins, which are effective in reducing free radicals, thus demonstrating significant health benefits [8]. However, red rice flour, like other gluten-free flours, has poor gas-holding capacity and low protein content, which is not beneficial for gluten-free noodle production. Fermentation treatments are commonly used to improve the texture and quality of gluten-free flour [9]. Lancetti et al. [10] used indigenous fermentation strains to ferment refined rice, quinoa, and buckwheat flours, respectively, and found that fermentation significantly altered flour pasting characteristics and increased flour antioxidant activity. Mudau et al. [11] found that spontaneous fermentation enhanced the nutritional value and health-promoting compounds of gluten-free millet crackers. *Lactobacillus plantarum*, an important species in the genus *Lactobacillus*, can produce enzymes and organic acids during fermentation, can modify the starch granules, and is beneficial to enhance the intermolecular interactions between starch molecules, making it easier for starch molecules to form an organized gel network [12]. Thus, fermentation using *Lactobacillus plantarum* may be one of the ways to enhance the viscoelasticity and texture of noodles [13].

Fava beans (*Vicia faba* L.) are rich in nutrients such as protein, dietary fiber, and minerals, which may positively impact chronic health problems [14]. However, fava beans contain antinutritional factors as secondary metabolites that reduce their protein digestibility and nutrient utilization [15]. Germination as a biological process is an effective and less costly processing method to mitigate or eliminate the negative effects of beans [16]. Lakshmipathy et al. [17] found that germination significantly increased the protein content and bioactivity of grass pea flour while decreasing the total carbohydrate and fat levels. Akkad et al. [18], in a study on the flavor quality of germinated fava beans, found that germination altered the headspace volatiles in the fava bean flour, and volatiles imparting a soya flavor were reduced after germination. Therefore, sprouted legumes became an important source of ingredients for new functional foods. However, few studies have been reported on the use of sprouted legumes and fermented red rice mixed flours in gluten-free products.

In summary, fermentation enhances flavor and enriches bioactive compounds, while sprouting optimizes nutritional composition. Therefore, this study combines fermented red rice flour with sprouted fava bean flour, investigates the functional, pasting, and rheological properties of germinated fava bean flour, *Lactobacillus plantarum* fermented red rice flour, and mixed flour. The effects of germination and fermentation on the structural properties of fava bean dough, red rice dough, and mixed dough, as well as the quality and functional properties of the mixed noodles, were then investigated. This study was purposed to develop novel gluten-free noodles with superior texture and nutritional profile, offering new insights for product innovation in related industries.

## 2. Materials and Methods

### 2.1. Materials

The red rice (Huiliangyou 1898) (moisture content 10.71%, crude protein 8.65%, ash 2.63%, total carbohydrates 75.30%, and fat 2.71%) was supplied by Anhui Echo Valley Agricultural Products Co., Ltd. (Anqing, China). The fava beans (White-skinned fava beans) (water content 10.36%, crude protein content 21.60%, ash content 3.14%, total carbohydrate 63.41%, and fat content 1.49%) were provided by Yunnan Siji Yuan Agricultural Technology Co., Ltd. (Kunming, China). *Lactobacillus plantarum* (JYLP326) was purchased from China Zhongke Jiayi Bioengineering Co. Reagents, including 1,1-diphenyl-2-picrylhydrazyl (DPPH), 2,2′-Azinobis-(3-ethylbenzthiazoline-6-sulphonate) (ABTS), α-amylase (4000 U/g), glucoamylase (100,000 U/g), and pepsin (3000 U/g) were obtained from Yuanye Biotechnology (Shanghai, China). The 3,5-dinitrosalicylic acid reagent (DNS) was obtained from Coolab Technology (Beijing, China). All additional chemicals of analytical grade were purchased from Sinopharm Chemical Reagent (Shanghai, China).

### 2.2. Pre-Treatment of Materials

#### 2.2.1. Fermentation Treatment of Red Rice

*Lactobacillus plantarum* was activated in de Man, Rogosa, and Sharpe (MRS) medium (at 37 °C for 24 h) to obtain a bacterial suspension (10^8^ CFU/mL). Red rice (100 g) used for fermentation was washed and placed in sterilized conical flasks and mixed with 200 mL of sterile water, while 2% (*v*/*w*, based on the weight of red rice) of the bacterial suspension was added. The mixture was then incubated at 37 °C for 48 h in a constant-temperature incubator (BSC-150, Boxun Instruments Co., Ltd., Suzhou, China). Humidity was maintained at 80%, and the mixture was gently stirred every four hours under sterile conditions to aerate it [19]. The pH of the mixture before and after fermentation was 6.43 ± 0.02 and 4.08 ± 0.02, respectively. The control group was unfermented red rice. The fermented and control red rice were washed three times with deionized water, and then oven-dried at 50 °C for 6 h. The dried red rice was pulverized and passed through an 80-mesh sieve to obtain fermented red rice flour (F-RRF) and raw red rice flour (RRF).

#### 2.2.2. Sprouting Treatment for Fava Beans

Fava bean seeds were treated with a sodium hypochlorite solution (0.7 g/100 g) for 30 min, then rinsed with deionized water until pH neutral and soaked in deionized water for 12 h. The soaked seeds were evenly spread on wet gauze and germinated at 25 °C and 80% relative humidity in the dark for 24 h. The germinated (seed germination rate was 97.3 ± 1.5%) and raw fava bean seeds (control) were oven-dried at 50 °C for 6 h. The dried fava beans were crushed and passed through an 80-mesh sieve to obtain sprouted fava bean flour (S-FBF) and control (FBF).

### 2.3. Preparation of Mixed Flour

S-FBF and F-RRF were mixed at different mass ratios (10%, 20%, 30%, 40%, and 50% S-FBF, *w*/*w*) and labeled as S-F10, S-F20, S-F30, S-F40, and S-F50, respectively.

### 2.4. Determination of Physiochemical Properties of Flours

#### 2.4.1. Water Absorption Capacity, Solubility, and Swelling Power

The water absorption capacity (WAC), solubility (SL), and swelling power (SP) of flours were determined using the method of Park et al. [20] with minor modifications. A 2% (*w*/*w*) suspension of the flours was shaken in a water bath at 95 °C for 30 min. The samples were cooled and centrifuged at 3000 g for 10 min. The supernatant was collected and dried at 105 °C for 4 h. The weight of the dried supernatants and the weight of the precipitates were then weighed separately. WAC, SL, and SP were calculated according to the following formula.

(1) $$WAC\left(g\;water/g\;sample\right)=W_{2}/W_{0}$$

(2) $$SL\left(\%\right)=W_{1}/W_{0}\times 100$$

(3) $$SP\left(g\;water/g\;insoluble\;residue\right)=\frac{W_{2}}{W_{0}\times \left(1-\frac{SL\left(\%\right)}{100}\right)}$$

where W_0_ is the weight of the flour, W_1_ is the weight of the dried supernatant, and W_2_ is the weight of the wet sediment.

#### 2.4.2. Thermal Properties

The thermal properties of the flour were determined using a differential scanning calorimeter (DSC Q20, TA Instruments, New Castle, DE, USA). The flour sample (3 mg) was added to deionized water in the ratio of 1:3 (*w*/*w*), sealed in a DSC crucible, and left overnight at room temperature. The heating was carried out from 25 °C to 120 °C at a rate of 10 °C/min using an empty crucible as a reference.

#### 2.4.3. Pasting Properties

The pasting properties of flour were determined by means of a rapid viscosity analyzer (TECMASTR, PerkinElmer Instruments Co., Ltd, Waltham, MA, USA). Referring to the method of Kang et al. [21], the flour sample (3 g) was weighed and dispersed into 25 mL of deionized water in an RVA aluminum canister. The sample suspension was kept at 50 °C for 1 min, then at 95 °C for 2.5 min, and finally at 50 °C for 2 min.

#### 2.4.4. Dynamic Rheological Measurements

Based on the method of Cai et al. [22], the viscoelasticity of the flour was analyzed using a rheometer (KINEXUS LAB+, Netzsch Group, Bavaria, Germany), with slight modifications. The flour sample (3 g) was weighed and dispersed in 25 mL of deionized water, mixed, and placed in a 90 °C water bath, and stirred for 15 min. The paste was equilibrated to room temperature prior to analysis. The samples were placed on a rheometer plate (50 mm diameter) at a constant temperature of 25 °C with a gap of 1 mm between the probe and the plate. Frequency scan tests were performed at 0.1–10 Hz with a constant strain value of 1%.

### 2.5. Preparation of Dough

FBF, S-FBF, RRF, F-RRF, and mixed flours were thoroughly mixed with 60% deionized water (*w*/*w*). The mixture was then processed into dough using a Joyoung mixer (MC530, Joyoung Company Limited, Jinan, China) at 70 rpm for 2 min. Its formula contains only two ingredients: flour and deionized water, with no added salt or other components. A portion of the dough sample was freeze-dried and ground to pass through an 80-mesh sieve for further analysis.

### 2.6. Interaction of the Main Components in the Dough

#### 2.6.1. Chemical Interactions

Chemical interactions were determined using a slightly modified method of Cao et al. [23]. The solvents were (1) 0.05 M NaCl (PA), (2) 0.6 M NaCl (PB), (3) 0.6 M NaCl + 1.5 M urea (PC), and (4) 0.6 M NaCl + 8 M urea (PD). Four different solvents were prepared in a 0.05 M pH 7.0 phosphate buffer. Freeze-dried flours (0.2 g) were added to 5 mL of the above reagents. After shaking at room temperature for 2 h, they were centrifuged at 4000 g for 20 min. Soluble protein content was determined using the Bradford method [24]. Ionic bonding was expressed as the difference between soluble proteins in PB and PA, hydrogen bonding was expressed as the difference between soluble proteins in PC and PB, and hydrophobic interactions were expressed as the difference between soluble proteins in PD and PC.

#### 2.6.2. X-Ray Diffraction (XRD)

X-ray diffractograms of freeze-dried dough samples were obtained using an X-ray diffractometer (SMARTLAB9, Rigaku Corporation, Tokyo, Japan) operating at 40 mA and 40 kV. The diffractograms were obtained in the range of 4–45° (2θ), at a scanning speed of 2°/min and a scanning step of 0.02°.

#### 2.6.3. Fourier Transform Infrared (FTIR) Spectroscopy

Spectral information of the samples was obtained using an FTIR spectrometer (NICOLET IS50, Thermo Fisher Scientific, Waltham, Massachusetts, USA). Freeze-dried flour was mixed with potassium bromide in the ratio of 1:100 and ground uniformly in an agate mortar, then pressed into thin pellets using a tablet press (SYP-2TH, Xin Nuo Instrument Equipment Co., Ltd., Shanghai, China). The scanning range was 400–4000 cm^−1^ with a resolution of 4 cm^−1^, and 32 scans were averaged for each spectrum. The spectra were analyzed using Omnic and Peakfit 4.12.

#### 2.6.4. Moisture Distribution Analysis

A low-field nuclear magnetic resonance analyzer (LMR-30, Larmor Technology Development Co., Ltd, Beijing, China) was used for testing the moisture distribution in the dough. The transverse relaxation time T2 was measured by scanning with a Carr–Purcell–Meiboom–Gill (CPMG) pulse sequence. Key parameters were set as follows: resonance frequency 7 MHz, temperature 32 ± 0.01 °C, echo time 0.15 ms, and number of sampling points 121,204. Number of echoes 2000, scan repetition set to 8 times.

### 2.7. Preparation of Noodles

The dough was prepared by thoroughly mixing the mixed flour with 60% deionized water (*w*/*w*), and the mixture was then processed into dough using a Joyoung mixer (MC530, Joyoung Company Limited, Jinan, China) at 70 rpm for 2 min. Then, it was placed on a food-grade tray and steamed at 100 °C for 10 min (ZGB2605, Joyoung Company Limited, Jinan, China) [25]. Next, the dough was extruded into 2 mm strips by using a noodle machine (M2-MS180, Joyoung Company Limited, Jinan, China).

### 2.8. Determination of Quality Properties of Noodles

#### 2.8.1. Microstructure of Noodles

(1) Scanning electron microscopy (SEM)

The microstructure of the cross-section of cooked noodles was observed using a scanning electron microscope (S4800, Hitachi, Ltd., Tokyo, Japan). The cooked noodle samples were freeze-dried and cut into 2 mm-long sections, fixed in a sample holder, sprayed with gold, and imaged at an accelerating voltage of 1 KV.

(2) Confocal laser scanning microscopy (CLSM)

The morphology of the protein network of cooked noodles was observed using CLSM (STELLARIS 5, Leica Company, Wetzlar, Germany). The cooked noodle samples were freeze-dried and sliced into 40 μm thick slices using a frozen slicer (CM1520, Leica Company, Wetzlar, Germany), then a mixture of rhodamine B (0.025% *w*/*v*) and FITC (fluorescein isothiocyanate) (0.25% *w*/*v*) dyes was dropped on the surface of the sliced freeze-dried noodle, and reacted for 15 min in the dark. The excitation wavelength for the rhodamine B was 568 nm, and the FITC excitation wavelength was 488 nm.

#### 2.8.2. Cooking Properties

Different samples of noodles were cut into identical lengths, and 10 noodles were weighed separately to record the pre-cooking mass (m_0_). Subsequently, the noodles were cooked in 500 mL of boiling water until the white centers disappeared. The cooked noodles were cooled in distilled water for 30 s and then placed on filter paper to drain the surface of the noodles to record the weight of the noodles after cooking (m_1_) and the number of broken noodles (N). The cooking water was collected and diluted to 500 mL with distilled water and dried in an oven at 105 °C until constant weight, and the mass of the broth behind the constant weight was weighed and recorded as (m_2_). The water absorption rate, cooking loss rate, and the breakage rate of noodles were calculated according to the following equation:

(4) $$Water\;absorption\left(\%\right)=\left(m_{1}-m_{0}\right)/m_{0}\times 100$$

(5) $$Cooking\;loss\left(\%\right)=m_{2}/m_{0}\times 100$$

(6) $$Breakage\;rate\left(\%\right)=N/10\times 100$$

#### 2.8.3. Textural Properties

The textural properties of cooked noodles were analyzed using a texture analyzer (CT3, Brookfield Engineering Laboratories Company, Massachusetts, Middleboro, USA). Three cooked noodles were placed next to each other on the test bench and subjected to two compression cycles using a P36/R probe at a trigger force of 5 g, a speed of 1 mm/s, and a compression ratio of 50%.

#### 2.8.4. Color

The color of cooked noodles was measured using a colorimeter (CR-400, Konica Minolta Holdings Inc., Tokyo, Japan), which was calibrated with a white standard plate before each use. The results are expressed as L* (0 = black, 100 = white), a* (+a* = red, −a* = green), b* (+b* = yellow, −b* = blue).

#### 2.8.5. Total Phenolic Content and Antioxidant Activity

The phenolic compounds were extracted using ethanol with slight modifications from the method described by Cai et al. [26]. Place the sample (1 g) into a centrifuge tube and add 15 mL of 80% ethanol. Incubate in an ultrasonic cleaner (Model 15A481, Ningbo Xinzhi Biological Co., Ltd., Ningbo, China) at 50 °C for 30 min, then centrifuge at 6500 rpm/min for 10 min. Collect the supernatant, repeat the process three times, and combine the supernatants to a final volume of 50 mL. The total phenolic content of the noodles was determined using the Folin–Ciocalteu reagent method [27]. In brief, 0.3 mL of extract was thoroughly vortexed with 2.5 mL of 0.2 mol/L Folin–Ciocalteu phenol reagent. After a 10 min reaction, 2 mL of 8% Na_2_CO_3_ solution was added. After 1 h of dark incubation at room temperature, reaction mixtures were spectrophotometrically analyzed at 725 nm (UV-1200S, Aoyi Instruments (Shanghai) Co., Ltd., Shanghai, China). Calculate the total phenolic content using the standard curve equation (y = 88.327x + 0.0541, R^2^ = 0.9991). The results were expressed as gallic acid equivalents per gram of dry weight of the sample (mg GAE/g dry weight).

DPPH free radical scavenging capacity was measured according to the method of González-Cervantes et al. [28], with slight modifications. A 2 mL portion of the extract was mixed with 2 mL of DPPH ethanol solution (0.2 mmol/L). The mixture was reacted in the dark at room temperature for 30 min. The absorbance was measured at 517 nm (UV-1200S, Aoyi Instruments Co., Ltd., Shanghai, China). Distilled water was used instead of the sample solution as a blank group, and it was used instead of the DPPH working solution as a control group. And the results were expressed using the following formula:

(7) $$DPPH\;scavenging\;activity\left(\%\right)=\left(1-\frac{{Abs}_{Sample}-{Abs}_{Control}}{{Abs}_{Blank}}\right)\times 100$$

ABTS free radical scavenging capacity was performed as described by Cai, Shen, Li, Xiong and Li [26] with slight modifications. We mixed 0.5 mL of the extract with 4 mL of ABTS working solution, and the reaction was carried out in the dark for 6 min at room temperature. The absorbance was measured at 734 nm (UV-1200S, Aoyi Instruments, Shanghai, China). Distilled water, instead of the sample solution, was used as the blank group, and distilled water, instead of the ABTS-working solution, was used as the control group.

(8) $$ABTS\;scavenging\;activity\left(\%\right)=\left(1-\frac{{Abs}_{Sample}-{Abs}_{Control}}{{Abs}_{Blank}}\right)\times 100$$

The hydroxyl radical scavenging capacity was determined by the method of Zhang et al. [29] with slight modifications. A 1 mL portion of the extracted solution was mixed with 0.5 mL of 9 mmol/L salicylic acid solution, 0.5 mL of 9 mmol/L FeSO_4_ solution, and 5 mL of 8 mmol/L hydrogen peroxide solution. The solution was reacted in the dark at room temperature for 6 min, and the absorbance was measured at 510 nm. Distilled water was used as a blank group instead of the sample solution, and distilled water was used as a control group instead of the FeSO_4_ solution.

(9) $$OH\;scavenging\;activity\left(\%\right)=\left(1-\frac{{Abs}_{Sample}-{Abs}_{Control}}{{Abs}_{Blank}}\right)\times 100$$

#### 2.8.6. In Vitro Digestibility of Starch

The in vitro digestion of starch was conducted using the method of Lin et al. [30] with some alterations. We dissolved 200 mg of noodle freeze-dried flour in 15 mL of acetic acid buffer solution (0.2 M, pH 5.6) and mixed in a boiling water bath for 30 min. After cooling, 5 mL of pepsin (1 mg/mL) was added and incubated at 37 °C for 30 min, and finally, 5 mL of a mixture of α-amylase (290 U/mL) and glucoamylase (50 U/mL) was added for the digestion reaction. At 0, 10, 20, 30, 60, 90, 120, and 180 min of digestion, 1 mL of the hydrolysis product was taken and mixed with 4 mL of 95% ethanol for enzyme inactivation. The glucose content was determined using 3,5-dinitrosalicylic acid (DNS). The percentage of fast digestible starch (RDS), slow digestible starch (SDS), and resistant starch (RS) was calculated using the following formula:

(10) $$RDS\left(\%\right)=\frac{{(G}_{20}-FG)\times 0.9}{TS}\times 100$$

(11) $$SDS\left(\%\right)=\frac{{(G}_{120}-G_{20})\times 0.9}{TS}\times 100$$

(12) $$RS\left(\%\right)=100\%-RDS\left(\%\right)-SDS\left(\%\right)$$

where FG and TS correspond to the free glucose content and total starch content of the samples, respectively. G_20_ and G_120_ denote the glucose content at 20 min and 120 min of hydrolysis, respectively.

A first-order kinetic model was used to calculate the simulated in vitro digestibility of the noodles at each digestion time:

(13) $$C_{t}=C_{\infty }\left(1-e^{-kt}\right)$$

where C_t_ is the starch digestibility at time t (min), C_∞_ is the estimated percentage of starch dissolution at the end of the reaction, and k is the starch dissolution rate coefficient.

The eGI of the noodles was calculated by the following equation:

(14) eGI=HI×0.862+8.198

Hydrolysis index (HI) is the ratio of the area under the hydrolysis curve (AUC) between the noodles and the white bread.

### 2.9. Statistical Analysis

All tests were performed in triplicate. All results are expressed as the mean ± standard deviation. Analysis of variance, Duncan test, and SPSS 26 software (SPSS Inc., Chicago, IL, USA) were used, and *p* < 0.05 was considered significant.

## 3. Results and Discussions

### 3.1. Water Absorption Capacity, Solubility, and Swelling Power Swelling

**Figure 1:**
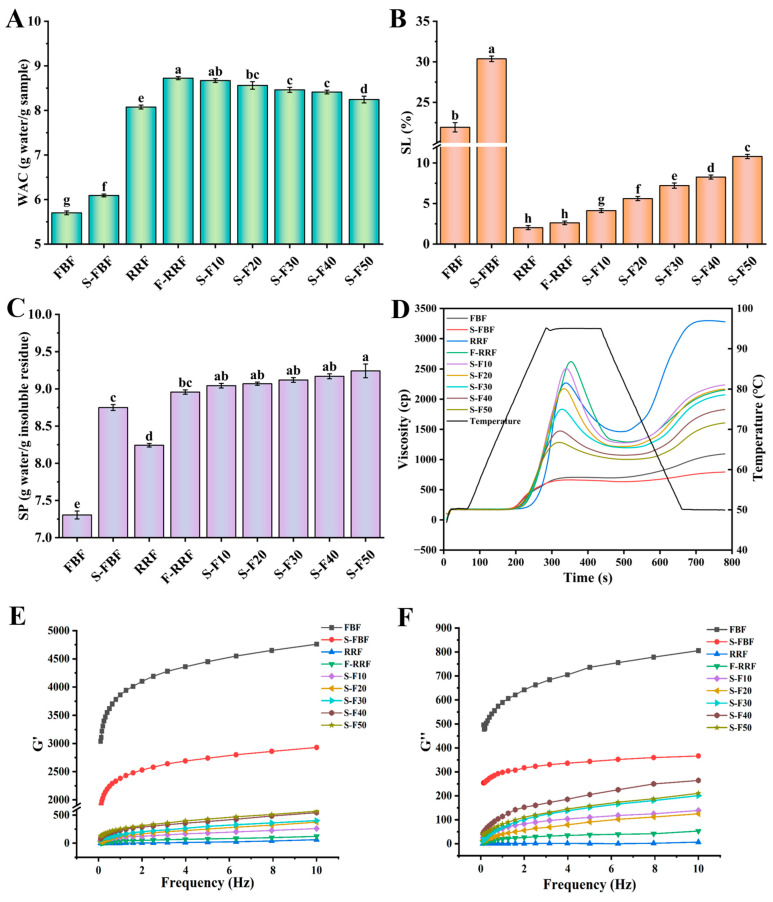
Hydration properties, pasting curves, and rheological properties of FBF, S-FBF, RRF, F-RRF, and mixed flours. (**A**) water absorption capacity (WAC), (**B**) solubility (SL), (**C**) swelling power (SP), (**D**) pasting curves (**E**) G′: energy storage modulus, and (**F**) G″: loss modulus. Different letters in the same group indicate significant differences (*p* < 0.05).

Changes in water absorption capacity, solubility, and swelling power of FBF, S-FBF, RRF, F-RRF, and mixed flours are shown in Figure 1A–C. Germination and fermentation treatments significantly increased the hydration capacity of the flours. This may be attributed to the fact that germination and fermentation treatments promote the uptake of water molecules by starch granules [31]. Swelling power indicates the extent to which starch absorbs water, while solubility measures its dissolution during the swelling process. During starch swelling, amylose constitutes the primary soluble component in the supernatant. The ratio of amylose to amylopectin, along with the molecular weights and distribution of these two starches, can influence the degree of this interaction, leading to variations in starch swelling properties and solubility [32]. The solubility and swelling power of the mixed flours showed an increasing trend as the percentage of S-FBF addition increased. Increased solubility of mixed flours may be associated with an increased content of straight-chain starch [33]. Previous studies have suggested that noodles with high rectilinear starch content were effective in reducing postprandial blood glucose concentration [34]. However, an increase in expansion force leads to a decrease in the densification of the network of noodles. Therefore, there is a need to balance the ratio of S-FBF and F-RRF in the production of noodles.

### 3.2. Thermal Properties

The DSC results of FBF, S-FBF, RRF, F-RRF, and mixed flours are shown in Table 1. Under the conditions studied, thermal transformations induced by starch pasting could be observed [35]. Germination reduces the temperature and energy required for the pasting of FBF. This may be related to the fact that germination causes a reduction in the fat layer around the starch granules in flour, which makes the granules of germinated flour more susceptible to thermal expansion during heating [36]. The results were in agreement with the findings of Sofi et al. [37] in the study of sprouted chickpea. At the same time, fermentation decreased the pasting temperature of RRF and increased the ΔH. It is noteworthy that the pasting temperature and ΔH of FBF were significantly lower than those of RRF, which suggests that FBF possesses a higher degree of pasting and a lower degree of crystallinity. The low ΔH value may be related to the dissociation of the double-helix structure within starch granules and the disruption of amorphous regions during the heating process [38]. The values of T_0_, T_p_, T_c_, and ΔH decreased gradually with the addition of S-FBF. These results show that the addition of S-FBF is able to inhibit starch retrogradation and maintain the elasticity of the noodles after cooking.

### 3.3. Pasting Property

The pasting curves of FBF, S-FBF, RRF, F-RRF, and mixed flours are shown in Figure 1D, and the pasting parameters are shown in Table 1. The pasting viscosity and temperature of S-FBF were reduced as compared to FBF. The results are in agreement with those reported by Sharma et al. [39] in germinated millet flour. Compared to RRF, F-RRF showed lower trough viscosity, final viscosity, setback, and paste temperature. The decrease in setback of colored brown rice after fermentation was also reported in the study of Wahyuni, Asnani, Khaeruni, Dewi, Sarinah and Faradilla [9], suggesting the ability of fermentation to improve the aging resistance of RRF. The setback of mixed flours decreased significantly (*p* < 0.05) with increasing S-FBF addition. Lower setback can reduce texture changes caused by starch aging, thus maintaining better taste and texture of the food [40]. These results showed that the addition of S-FBF could enhance the viscosity stability of the mixed system, while reducing the temperature required for pasting and accelerating the pasting process, improving the adaptability of the noodles for processing.

### 3.4. Dynamic Rheological Measurements

The effect of the rheological properties of FBF, S-FBF, RRF, F-RRF, and mixed flours is shown in Figure 1E,F. FBF showed a decreasing trend in both G′ and G″ after germination. Both G′ and G″ of the mixed flours showed an increasing trend with increasing S-FBF addition, and G′ > G″, which suggests that the mixed flours exhibited pseudoplastic behavior. Similar results were obtained in the study of Sofi et al. [41]. In addition, each sample underwent a heating and cooling process prior to the rheological characterization tests. Therefore, G′ and G″ can be considered as indicators of starch aging [22]. These results indicate that the addition of S-FBF increases the gel strength of the mix, which promotes bonding between the starch granules of the dough.

### 3.5. Chemical Interactions

**Figure 2:**
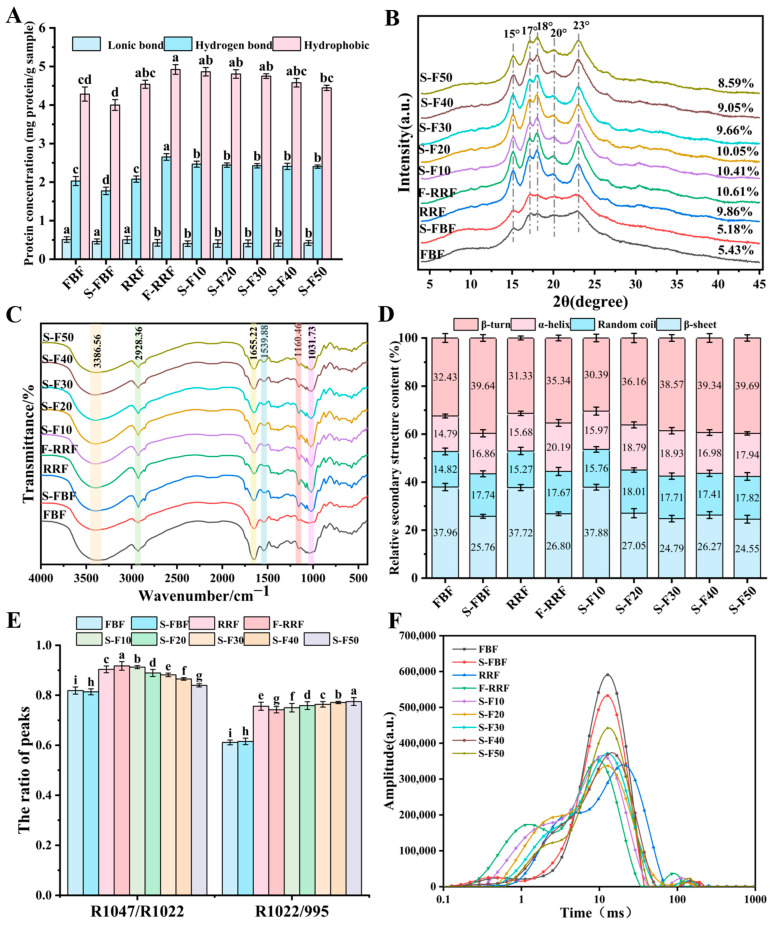
(**A**) Chemical interaction forces, (**B**) XRD patterns, (**C**) FTIR spectra, (**D**) relative composition of protein secondary structures, (**E**) 1047/1022 and 1022/995 ratios of short-range ordered structures of starch, (**F**) moisture distribution properties of FBF, S-FBF, RRF, F-RRF, and their mixed doughs, respectively. Different letters in the same group indicate significant differences (*p* < 0.05).

The chemical interaction forces between starch and protein in dough are shown in Figure 2A. The contribution of hydrophobic interactions to the starch–protein interactions in the dough was more significant than that of hydrogen and ionic bonds [42]. In F-RRF dough, ionic bond strength was weakened, and hydrogen bond strength was enhanced, a phenomenon that may be related to the abundance of hydroxyl and amino-hydrogen bonding sites provided by peptides produced by protein hydrolysis due to fermentation. Changes in the proportion of mixed flours had no significant effect on the ionic and hydrogen bond strengths, but the hydrophobic interactions were gradually weakened, which may be related to the fact that the complex formed by fava bean protein and red rice starch masked the hydrophobic groups. The formation of the dough network structure is associated with protein molecular aggregation, which affects protein structural stability [43].

### 3.6. X-Ray Diffraction (XRD)

Figure 2B shows the XRD patterns of FBF and S-FBF, RRF and F-RRF, and mixed flours. The diffraction patterns showed diffraction peaks at 15°, 17°, 18°, 20°, and 23° (2θ) for the different flour samples, indicating that the crystal type of starch is an A-type structure. The positions of the diffraction peaks were not significantly shifted, indicating that the crystalline structure of the starch was not altered due to germination, fermentation, and mixing treatments. The relative crystallinity of S-FBF decreased from 5.43% to 5.18%. The relative crystallinity of F-RRF increased from 9.86% to 10.61%. Similar conclusions were obtained by Lu et al. [44] in their study of the effect of natural fermentation on the crystalline structure of whole refined rice grains. As the proportion of S-FBF added increased, the relative crystallinity of the mixture decreased from 10.61% to 8.59%, and the intensity of A-type diffraction peaks gradually weakened. The results were consistent with the findings in the study of Liu et al. [45]. These results suggest that the addition of S-FBF reduced the crystallinity of the mixed dough, which may contribute to the reduction in the glycemic index and facilitate the production of functional F-RRN.

### 3.7. FTIR Analysis

#### 3.7.1. Functional Group Structure Analysis

FTIR spectra of FBF, S-FBF, RRF, and F-RRF, and mixed flours in the range of 4000–400 cm^−1^ are shown in Figure 2C. The morphology of the spectrograms was similar for all the samples, but differences in the intensity of the absorption peaks could be observed. The absorption peaks around 3400–3200 cm^−1^ observed in different flour samples were attributed to O-H stretching vibrations, reflecting the hydration of dough-bound water molecules [46]. The absorption peaks at around 2850–2950 cm^−1^ were induced by lipids in response to the amylose C-H stretching vibrational absorption peaks of lipid complexes [47]. The absorption peaks in the range of 1700–1600 cm^−1^ could be attributed to the C=O stretching in the amide I region, whereas the absorption peaks in the range of 1600–1500 cm^−1^ could be attributed to the N-H bending and C-N stretching in the amide II region. The absorption peaks around 2850–2950 cm^−1^ were attributed to lipid-induced C-H stretching vibrations. The absorption peaks around 1200–1000 cm^−1^ were attributed to C-O/P=O stretching vibrations caused by polysaccharides or phosphoric acid. As the proportion of S-FBF increased, the absorption intensity of the mixed flours in this region gradually weakened, indicating a certain tendency of structural reorganization of polysaccharides or small polar molecules in the composite system. Figure 2C shows that the starch-protein interactions of the treated mixed dough can reach equilibrium.

#### 3.7.2. Protein Secondary Structure Analysis

The secondary structure of the proteins of the dough is shown in Figure 2D. FTIR analysis of protein conformations in samples was conducted by examining the amide I region (1700–1600 cm^−1^) to determine protein secondary structure. Iterative fitting based on a Gaussian distribution, combined with second-derivative analysis to locate absorption band centers, estimated the secondary structural composition of proteins based on relative band areas: β-turns (1700–1660 cm^−1^), α-helix (1658–1650 cm^−1^), random coil (1650–1640 cm^−1^), and β-sheet (1640–1600 cm^−1^) [48]. From a single treatment, the β-sheet content in germinated and fermented doughs decreased, while β-turns, α-helices, and random coils increased, indicating that the germination and fermentation processes induced the formation of helices and flexible structures. In the mixed doughs, the β-sheet content decreased from 37.88% to 24.55% with increasing S-FBF addition, while the β-turns content increased from 30.39% to 39.69%, and α-helices and random coils showed a slight increase throughout the fluctuations. Differences in the secondary structure of proteins in doughs may be due to differences in the conformation of proteins in sprouted and fermented flour, which may affect the rheological behavior of doughs [49].

#### 3.7.3. Analysis of Short-Range Ordering of Starch

The absorption peak intensity ratio of (1047/1022) cm^−1^ versus (1022/995) cm^−1^ is an index to evaluate the orderliness of starch structure and the stability of the amorphous region [50]. Figure 2E showed that the fermentation treatment significantly enhanced the ordered structure of the red rice starch but led to a decrease in the stability of the amorphous region arrangement. With the increase in S-FBF addition, the ordering of the crystalline region of the samples gradually decreased, while the ordering of the amorphous region was gradually enhanced, indicating that the structural softening effect of the germinated component gradually dominated the compound system, which might be beneficial for the adaptability of noodle processing.

Overall, although sprouting and fermentation treatments may weaken the intermolecular stabilization of proteins in dough, they have little effect on noodle processing.

### 3.8. Moisture Distribution Analysis

The low-field NMR relaxation time distribution curves of different doughs are shown in Figure 2F, and the water distribution parameters are shown in Table 2. Three types of peaks were observed in different dough samples, representing tightly bound water T21, less tightly bound water T22, and free water T23 [51]. The lower T21 and T23 values for S-FBF doughs compared to FBF doughs suggest that germination promotes rapid migration of dough moisture, which may contribute to improved dough processing properties. After fermentation, the T21, T22, and T23 values of RRF dough were shortened to 1.24 ms, 9.75 ms, and 89.33 ms, respectively, indicating that the mobility of water molecules in the dough decreased. The addition of S-FBF reduced the A23 value of the mixed dough to some extent. These results suggest that the addition of S-FBF can increase the amount of water bound in the mixed dough to some extent, which may be beneficial for the molding stability of dough.

### 3.9. Microstructure of Noodles

#### 3.9.1. SEM Analysis

**Figure 3:**
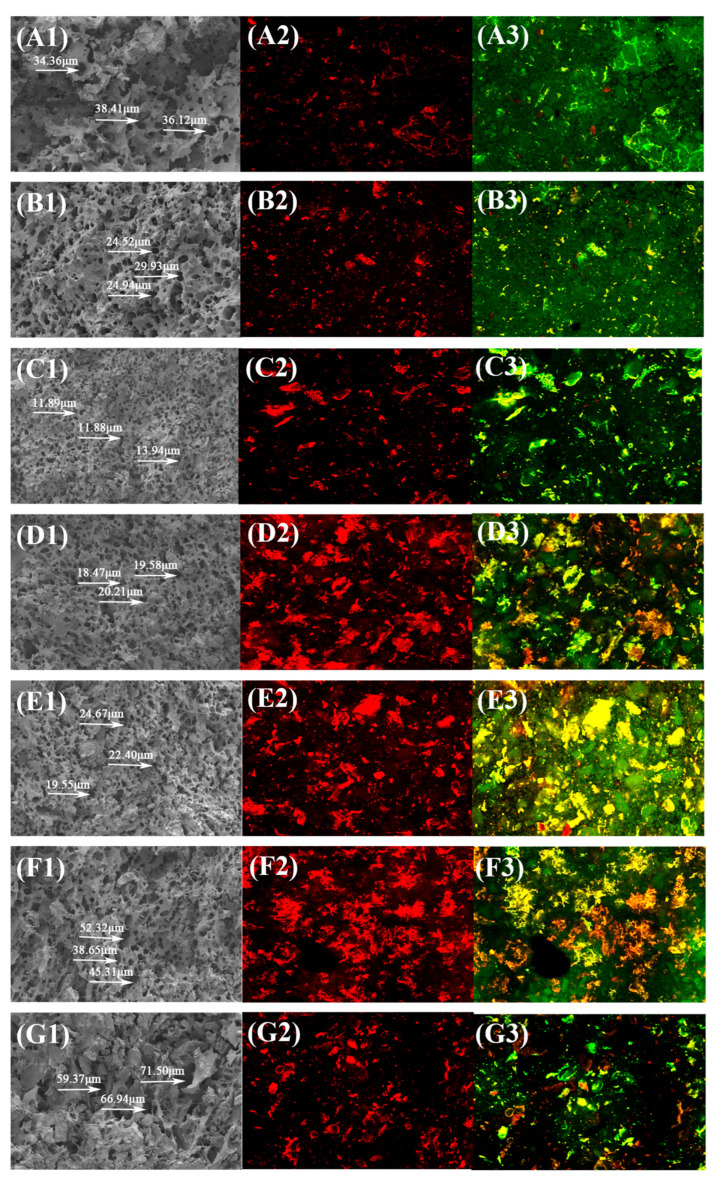
(**A1**–**G1**) illustrate SEM images of the cross-section of the cooked noodles. (**A2**–**G2**) illustrate the protein network structure of cooked noodles, while (**A3**–**G3**) illustrate their overall cross-sectional structure. SEM (1) 200× magnification and CLSM (2, 3) 50× magnification images of different noodle samples. (**A**–**G**) represent red rice noodles, fermented red rice noodles, and mixed noodles with 10–50% addition of S-FBF, respectively.

Figure 3(A1–G1) illustrates SEM images of the cross-section of the cooked noodles. As can be seen from the figure, the cross-section of the noodles showed a honeycomb structure, with larger and unevenly distributed pores in RRN; the pore size was 36.22 ± 2.10 μm. The pores in F-RRN were relatively smaller and more uniformly distributed, with a pore size of 26.46 ± 3.07 μm. The pores of S-F10 were significantly less than those of the other noodles, with a pore size of 12.57 ± 1.19 μm, suggesting that a dense protein network structure was formed. S-F30 still retained a relatively tight honeycomb structure. In contrast, S-F50 lost its structural integrity (the pore size is 65.94 ± 6.10 μm). These results showed that the addition of S-FBF was able to improve the quality of noodles to a certain extent.

#### 3.9.2. CLSM Analysis

CLSM was used to observe the protein network in Figure 3(A2–G2) and the slice structure in Figure 3(A3–G3) of noodles. The red region represents the protein network bound to rhodamine B, and the green region represents the starch granules bound to fluorescein isothiocyanate. Figure 3(A2–G2) showed that fermentation and the addition of S-FBF resulted in a more homogeneous and compact distribution of the protein network. Figure 3(C3–G3) shows that with the addition of S-FBF, the protein network structure was gradually developed, and the starch granules were distributed in the protein network, with some of them overlapping to some extent. The starch granules of S-F50 became loosely bound to the protein network. These results further indicate that adding S-FBF can improve the connectivity of the noodle protein network to a certain extent, which might contribute to the better quality of the noodles. However, adding high levels of S-FBF disrupts the protein network structure and adversely affects the quality of the final product.

### 3.10. Cooking Properties

Cooking properties of different noodles are shown in Table 3. Water absorption rate, cooking loss rate, and breakage rate are key parameters for evaluating noodle quality. Among these, the cooking loss rate directly reflects the degree of soup cloudiness caused by solid substances (such as starch and protein) leaching during noodle cooking; a higher loss rate indicates greater solid matter leaching and poorer noodle quality. The water absorption of F-RRN increased from 49.7% to 55.8% compared to RRN and decreased with the addition of S-FBF. This was attributed to the addition of S-FBF, weakening the binding of the mixture to water [52]. The cooking loss of F-RRN was reduced from 8.15% to 3.48%. This could be attributed to the fact that fermentation led to the formation of a more stable gel structure, which reduced the cooking loss. Although there was an increase in the cooking loss of the noodles with the addition of S-FBF, all of them were within the acceptable limit of cooking loss (12%) [53]. After fermentation, the breakage rate of noodles was reduced from 31.67% to 10.53%, indicating that fermentation was effective in improving the cooking quality of noodles. Although the addition of S-FBF increased the breakage rate of noodles, the breakage rate of the noodles was still lower than that of RRN in the range of 40% of the added amount.

### 3.11. Textural Properties

Textural properties are one of the primary factors affecting the overall quality of noodles. The texture quality of the noodles is shown in Table 3. High-quality noodles typically appear firm, springy, and chewy without being overly sticky [54]. After fermentation, the hardness, cohesiveness, and springiness of F-RRN were significantly higher than those of RRN. This may be due to protein denaturation caused by fermentation, as well as changes in the network interactions between starch and protein [20]. The hardness of the noodles increased with the addition of S-FBF, and the viscosity decreased, indicating that the addition of S-FBF made F-RRN smoother. This may be because the addition of S-FBF supports the internal structure of the noodles and forms a more robust gel structure [26]. The cohesion of the noodles with the addition of S-FBF decreased slightly compared to F-RRN, and the lowest cohesion was observed for S-F50, indicating that high additions of S-FBF resulted in a looser structure of the noodles. The maximum elasticity was observed for F-RRN, and there was no significant difference in elasticity between the noodles and the elasticity of F-RRN when the addition of S-FBF was in the range of 40%. Han et al. [55] reported that adding four different proportions of white kidney bean flour (10–40%) to wheat flour for noodle production resulted in increased hardness of cooked noodles, along with decreased cohesiveness and viscosity. These findings suggest that the addition of S-FBF in the range of 10–30% can effectively achieve the synergistic optimization of the textural parameters of noodles and improve the noodle quality.

### 3.12. Color Parameters of Noodles

The color parameters of the noodles are shown in Table 3. The L* of F-RRN was significantly higher, while a* and b* were significantly lower compared to RRN, indicating that fermentation could improve the brightness of the noodles. This may be due to lactic acid fermentation inhibiting Maillard browning by lowering the pH [19]. The L* and b* values of the noodles increased with the increase in the amount of S-FBF added, and the a* values gradually decreased. These differences may be related to the lighter color of S-FBF itself, while simultaneously producing a brightening effect on the color of the mixed noodles as the amount of S-FBF added increases. The addition of S-FBF effectively increased the brightness of the RRN, contributed to enhanced consumer acceptance, and showed greater potential for application in gluten-free food formulations.

### 3.13. Total Phenolic Content and Antioxidant Properties of Noodles

**Figure 4:**
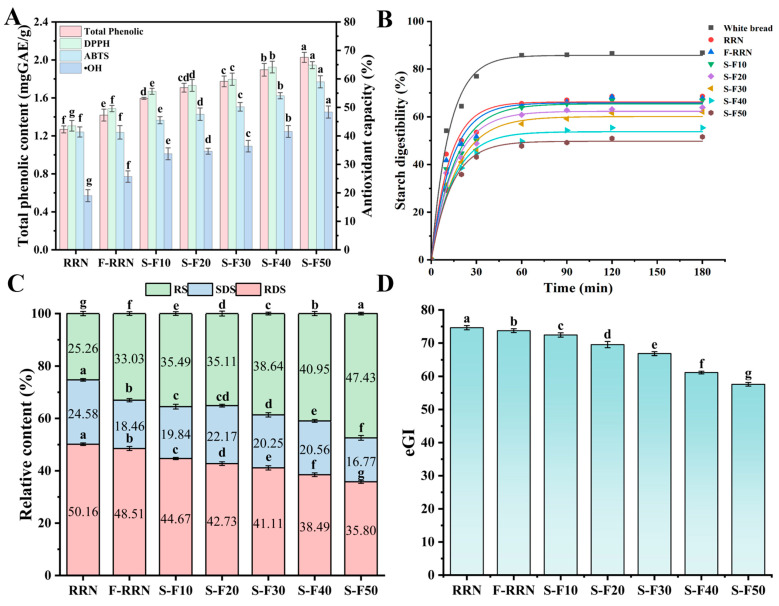
(**A**) Total phenolic content and antioxidant properties, (**B**) in vitro starch digestibility profiles, (**C**) RDS, SDS, RS content, and (**D**) eGI of different noodle samples. Different letters in the same group indicate significant differences (*p* < 0.05).

The results of total phenol content and antioxidant activity of different noodle samples are shown in Figure 4A. Studies indicated that microbial fermentation (including *Bacillus subtilis*, *Lactobacillus*, and *Saccharomyces cerevisiae*) effectively increased the total phenolic content and antioxidant activity of soybeans, mung beans, and other grains [56,57,58]. This can be explained by the production of metabolic byproducts (including bioactive substances, organic acids, etc.) through fermentation and the release of phenolic compounds from the food matrix [59]. In addition, fermentation processing may lead to an increase in phenolic compounds levels, such as anthocyanins, flavonoids, phenolic acids, and tannins, which directly enhance the antioxidant activity of the samples [59]. The total phenol content of RRN increased to 1.42 mg/g after fermentation, suggesting that fermentation promotes the release of polyphenols and enhances antioxidant activity [60]. In addition, the total phenolic content of the noodle samples increased significantly with increasing addition of S-FBF, especially reaching a maximum value of 2.03 mg/g at 50% addition of S-FBF, which was 59.84% higher than the RRN. This may be related to the fact that fava beans are rich in phenolics, while the germination process may activate phenolic precursors and increase the content of phenolic compounds [61].

Both DPPH radical scavenging capacity and OH radical scavenging capacity of F-RRN were significantly higher (*p* < 0.05) compared to RRN. The DPPH, ABTS, and OH radical scavenging of the noodles increased significantly with the addition of S-FBF, and reached the maximum at 50% S-FBF addition, which increased by 48.90%, 42.56%, and 154.63%, respectively, as compared to the control group. In conclusion, fermentation combined with the addition of S-FBF can synergistically enhance the antioxidant activity of RRN, and its effect is closely related to the effect of total phenolic content.

### 3.14. Starch Digestion and Glycemic Index

The starch digestibility in vitro curves, the RDS, SDS, RS contents, and the estimated glycemic index eGI values were shown in Figure 4B–D. From the in vitro starch digestibility curve, it can be seen that the starch digestibility increased rapidly in the first 20 min and increased slowly after 20 min to 180 min. F-RRN showed a significant decrease in RDS and SDS, a significant increase in RS, and a decrease in eGI from 74.64 to 73.74 when compared to RRN. This may be due to the fact that fermentation promotes the formation of resistant starch, which results in a lower eGI [12]. Compared with RRN, the RDS and SDS of the mixed noodle were significantly reduced, and RS was significantly increased with the increase in the addition of S-FBF, while the eGI of the mixed noodle was reduced. The decrease in the eGI may be related to the increase in the RS content with the addition of S-FBF. In conclusion, the addition of S-FBF can develop gluten-free noodles with relatively low eGI. It is worth noting that in vitro eGI aids in predicting the potential glycemic response of foods within the body, but it cannot fully replicate the complex processes of human digestion and metabolism [62]. In the future, we will conduct in vivo experiments to further validate these bioactivity results.

### 3.15. Pearson’s Correlation Coefficient Analysis

**Figure 5:**
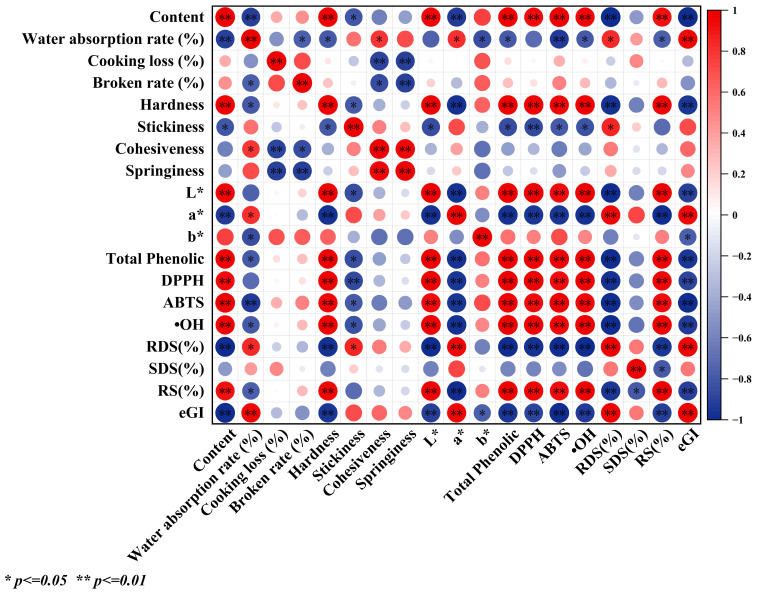
Heat map of Pearson correlation between different noodle samples in terms of addition of sprouted faba bean flour, cooking quality, texture properties, color, total phenolic content, antioxidant properties, and starch digestibility. Significance level. * *p* < 0.05, ** *p* < 0.01.

Pearson correlation analysis is shown in Figure 5. The addition of S-FBF showed a significant positive correlation with hardness, L* value, total phenol content, antioxidant properties, and RS content of noodles. Cooking loss showed a significant negative correlation with cohesiveness and springiness. Breakage rate showed a significant negative correlation with cohesiveness and springiness. The total phenol content showed significant positive correlation with antioxidant properties and RS content, and significant negative correlation with RDS content and eGI. These results showed that the addition of S-FBF had a positive effect on the quality of noodles. However, higher additions increased the cooking loss rate and breakage rate of noodles, which had a negative impact on consumer acceptability and was not favorable for the practical application of the product. Therefore, it is crucial to find the right amount of S-FBF to be added in the production of noodles.

## 4. Conclusions

In this study, we investigated the modifying effects of S-FBF, F-RRF, and the synergistic mechanism of the mixtures, and revealed their effects on the quality of gluten-free noodles. The results showed that the germination and fermentation treatments enhanced the processing adaptability of flour and dough, effectively improving the textural and functional properties of noodles. However, the addition of more than 40% S-FBF led to the loosening of the dough protein network, which resulted in an increase in the cooking loss rate and strip breakage rate of the noodles. Overall, low additions of S-FBF (10–30%) were effective in improving the nutritional and quality requirements of noodles, while high additions (40–50%) showed the opposite effect. This study provides a theoretical basis for the development of better acceptable gluten-free noodles. However, this study was only conducted in vitro antioxidant and glycemic index tests, lacking in vivo trials, as well as sensory quality analysis of products. Therefore, future research should incorporate in vivo experiments to evaluate its biological activity, including antioxidant and hypoglycemic activity, while conducting detailed sensory and flavor analyses for assessing overall sensory quality. These studies will enable a more comprehensive assessment of the product’s potential for development as a functional food, determining its practical application value and market prospects.

## Figures and Tables

**Table 1:** Thermal and pasting characteristics of FBF, S-FBF, RRF, F-RRF, and their mixed flours.

Samples	Thermal Properties				Pasting Property						
	T_0_ (°C)	T_p_ (°C)	T_c_ (°C)	ΔH (J/g)	Peak Viscosity (cP)	Trough Viscosity (cP)	Breakdown (cP)	Final Viscosity (cP)	Setback (cP)	Peak Time (min)	Paste Temperature (°C)
FBF	61.88 ± 0.45 ^e^	73.18 ± 0.65 ^h^	81.76 ± 1.09 ^h^	3.36 ± 0.22 ^g^	703.00 ± 3.61 ^h^	694.00 ± 3.46 ^h^	46.67 ± 3.06 ^h^	1073.67 ± 19.60 ^g^	379.67 ± 16.50 ^f^	6.80 ± 0.07 ^a^	82.08 ± 0.46 ^cd^
S-FBF	60.83 ± 0.57 ^i^	70.90 ± 0.78 ^i^	76.72 ± 0.87 ^i^	2.48 ± 0.34 ^h^	667.33 ± 5.86 ^i^	634.33 ± 4.16 ^i^	33.00 ± 2.65 ^h^	813.67 ± 20.03 ^i^	222.67 ± 4.04 ^g^	6.31 ± 0.23 ^b^	79.90 ± 0.05 ^f^
RRF	69.41 ± 0.79 ^a^	82.25 ± 0.55 ^a^	90.59 ± 0.67 ^a^	5.22 ± 0.19 ^e^	2221.50 ± 10.61 ^c^	1495.50 ± 3.54 ^a^	726.00 ± 14.14 ^d^	3290.00 ± 1.41 ^a^	1794.50 ± 4.95 ^a^	5.64 ± 0.05 ^de^	87.63 ± 0.53 ^a^
F-RRF	65.88 ± 0.64 ^b^	79.99 ± 0.70 ^b^	89.44 ± 0.64 ^b^	8.03 ± 0.35 ^a^	2624.50 ± 2.12 ^a^	1309.50 ± 27.58 ^b^	1315.00 ± 25.46 ^a^	2176.00 ± 38.18 ^c^	866.50 ± 10.61 ^c^	5.90 ± 0.04 ^c^	83.22 ± 0.11 ^b^
S-F10	63.17 ± 0.61 ^c^	79.49 ± 0.41 ^c^	88.86 ± 0.49 ^c^	6.00 ± 0.27 ^b^	2503.67 ± 9.24 ^b^	1289.00 ± 11.53 ^c^	1214.67 ± 15.31 ^b^	2238.00 ± 14.73 ^b^	956.00 ± 2.00 ^b^	5.71 ± 0.03 ^d^	82.95 ± 0.48 ^b^
S-F20	62.58 ± 0.78 ^d^	78.75 ± 0.49 ^d^	88.06 ± 0.75 ^d^	5.75 ± 0.33 ^c^	2187.00 ± 11.36 ^d^	1217.00 ± 7.21 ^d^	970.00 ± 12.77 ^c^	2173.33 ± 7.02 ^c^	953.33 ± 2.08 ^b^	5.55 ± 0.04 ^def^	82.63 ± 0.49 ^bc^
S-F30	61.72 ± 0.72 ^f^	78.35 ± 0.86 ^e^	87.70 ± 0.32 ^e^	5.55 ± 0.21 ^d^	1822.00 ± 12.00 ^e^	1184.67 ± 10.60 ^e^	637.33 ± 10.02 ^e^	2061.00 ± 10.00 ^d^	876.33 ± 1.53 ^c^	5.47 ± 0.00 ^efg^	82.05 ± 0.52 ^cd^
S-F40	61.36 ± 0.36 ^g^	77.98 ± 0.69 ^f^	86.86 ± 0.59 ^f^	5.23 ± 0.37 ^e^	1469.00 ± 5.66 ^f^	1074.50 ± 3.54 ^f^	394.50 ± 9.19 ^f^	1818.50 ± 12.02 ^e^	755.00 ± 15.56 ^d^	5.40 ± 0.00 ^fg^	81.58 ± 0.35 ^d^
S-F50	61.06 ± 0.72 ^h^	77.64 ± 0.41 ^g^	86.32 ± 0.83 ^g^	5.01 ± 0.31 ^f^	1277.00 ± 18.52 ^g^	1002.33 ± 5.13 ^g^	274.67 ± 14.43 ^g^	1608.33 ± 12.34 ^f^	606.00 ± 7.21 ^e^	5.33 ± 0.07 ^g^	80.72 ± 0.08 ^e^

Means followed by different letters in each column of the table indicate statistical differences (*p* < 0.05).

**Table 2:** Relaxation time T2 and peak area A2 of FBF, S-FBF, RRF, F-RRF, and mixed doughs.

Samples	T21 (ms)	T22 (ms)	T23 (ms)	A21 (%)	A22 (%)	A23 (%)
FBF	0.52 ± 0.04 ^g^	12.75 ± 0.05 ^d^	191.63 ± 0.15 ^b^	4.01 ± 0.61 ^i^	95.71 ± 0.21 ^a^	0.28 ± 0.61 ^h^
S-FBF	0.50 ± 0.17 ^g^	12.75 ± 0.05 ^d^	181.63 ± 0.21 ^c^	4.08 ± 0.39 ^h^	95.48 ± 0.64 ^b^	0.44 ± 0.25 ^g^
RRF	4.80 ± 0.04 ^a^	19.65 ± 0.03 ^a^	251.60 ± 0.44 ^a^	27.08 ± 0.19 ^d^	72.88 ± 0.25 ^f^	0.04 ± 0.63 ^i^
F-RRF	1.24 ± 0.05 ^f^	9.75 ± 0.02 ^f^	89.33 ± 0.35 ^i^	32.52 ± 0.98 ^a^	65.47 ± 0.47 ^i^	2.01 ± 0.56 ^a^
S-F10	1.95 ± 0.04 ^e^	10.83 ± 0.03 ^e^	117.63 ± 0.25 ^h^	30.44 ± 0.68 ^b^	68.21 ± 0.26 ^h^	1.35 ± 0.36 ^b^
S-F20	2.65 ± 0.02 ^c^	12.75 ± 0.05 ^d^	124.43 ± 0.42 ^g^	27.41 ± 0.75 ^c^	71.86 ± 0.47 ^g^	0.73 ± 0.85 ^f^
S-F30	2.67 ± 0.02 ^c^	12.75 ± 0.05 ^d^	131.17 ± 0.12 ^f^	21.47 ± 0.34 ^e^	77.59 ± 0.26 ^e^	0.94 ± 0.25 ^d^
S-F40	2.89 ± 0.08 ^b^	14.20 ± 0.05 ^b^	154.37 ± 0.15 ^d^	21.35 ± 0.35 ^f^	77.86 ± 0.96 ^d^	0.79 ± 0.86 ^e^
S-F50	2.51 ± 0.03 ^d^	13.47 ± 0.05 ^c^	138.41 ± 0.15 ^e^	15.27 ± 0.21 ^g^	83.71 ± 0.51 ^c^	0.99 ± 0.31 ^c^

Mean values in each column followed by different letters indicate statistical differences (*p* < 0.05).

**Table 3:** Cooking properties, textural properties, and color properties of noodles prepared from red rice flour.

Samples	Cooking Properties			Texture Properties				Color Properties		
	Water Absorption Rate (%)	Cooking Loss (%)	Breakage Rate (%)	Hardness (N)	Stickiness (mJ)	Cohesiveness	Springiness (mm)	L*	a*	b*
RRN	49.70 ± 0.36 ^bc^	8.15 ± 0.36 ^a^	31.67 ± 2.89 ^b^	15.17 ± 2.57 ^e^	0.09 ± 0.02 ^ab^	0.42 ± 0.10 ^b^	0.36 ± 0.02 ^b^	38.85 ± 1.44 ^f^	12.35 ± 0.14 ^a^	13.53 ± 0.22 ^ab^
F-RRN	55.80 ± 2.31 ^a^	3.48 ± 0.40 ^e^	10.53 ± 5.27 ^c^	21.17 ± 1.15 ^d^	0.10 ± 0.04 ^a^	0.76 ± 0.16 ^a^	0.50 ± 0.10 ^a^	41.21 ± 0.43 ^e^	10.83 ± 0.31 ^b^	12.82 ± 0.24 ^bc^
S-F10	53.00 ± 2.63 ^ab^	4.59 ± 0.13 ^d^	11.54 ± 0.38 ^c^	23.17 ± 1.61 ^cd^	0.06 ± 0.01 ^b^	0.60 ± 0.09 ^ab^	0.46 ± 0.05 ^a^	43.47 ± 0.00 ^d^	10.36 ± 0.06 ^bc^	12.55 ± 0.57 ^c^
S-F20	51.70 ± 1.57 ^abc^	5.97 ± 0.15 ^c^	12.28 ± 3.04 ^c^	26.00 ± 2.65 ^c^	0.05 ± 0.02 ^b^	0.52 ± 0.10 ^ab^	0.44 ± 0.03 ^ab^	44.10 ± 1.55 ^d^	10.24 ± 0.17 ^c^	12.87 ± 0.06 ^bc^
S-F30	47.33 ± 3.79 ^c^	6.57 ± 0.23 ^bc^	13.73 ± 6.8 ^c^	30.17 ± 1.26 ^b^	0.05 ± 0.01 ^b^	0.53 ± 0.02 ^ab^	0.42 ± 0.04 ^ab^	44.86 ± 0.25 ^cd^	10.01 ± 0.38 ^c^	13.86 ± 0.32 ^a^
S-F40	39.15 ± 3.79 ^d^	6.44 ± 0.44 ^bc^	21.69 ± 14.61 ^bc^	34.83 ± 1.15 ^a^	0.05 ± 0.02 ^b^	0.48 ± 0.05 ^ab^	0.42 ± 0.05 ^ab^	46.76 ± 0.95 ^bc^	8.50 ± 0.10 ^d^	14.17 ± 0.03 ^a^
S-F50	32.37 ± 2.10 ^e^	6.88 ± 0.24 ^b^	40.29 ± 3.21 ^a^	35.67 ± 1.53 ^a^	0.05 ± 0.02 ^b^	0.34 ± 0.33 ^b^	0.35 ± 0.03 ^b^	47.43 ± 0.74 ^b^	7.64 ± 0.37 ^e^	14.05 ± 0.51 ^a^

Different letters in the table indicate significant differences (*p* < 0.05).

## Data Availability

The original contributions presented in the study are included in the article; further inquiries can be directed to the corresponding authors.
